# Impact of Diverse Data Sources on Computational Phenotyping

**DOI:** 10.3389/fgene.2020.00556

**Published:** 2020-06-03

**Authors:** Liwei Wang, Janet E. Olson, Suzette J. Bielinski, Jennifer L. St. Sauver, Sunyang Fu, Huan He, Mine S. Cicek, Matthew A. Hathcock, James R. Cerhan, Hongfang Liu

**Affiliations:** ^1^Division of Digital Health Sciences, Department of Health Sciences Research, Mayo Clinic, Rochester, MN, United States; ^2^Division of Epidemiology, Department of Health Sciences Research, Mayo Clinic, Rochester, MN, United States; ^3^Center for Individualized Medicine, Mayo Clinic, Rochester, MN, United States; ^4^Division of Experimental Pathology, Department of Laboratory Medicine and Pathology, Mayo Clinic, Rochester, MN, United States; ^5^Division of Biomedical Statistics and Informatics, Department of Health Sciences Research, Mayo Clinic, Rochester, MN, United States

**Keywords:** phenotyping algorithms, computational phenotyping, rheumatoid arthritis, type 2 diabetes mellitus, diverse data sources

## Abstract

Electronic health records (EHRs) are widely adopted with a great potential to serve as a rich, integrated source of phenotype information. Computational phenotyping, which extracts phenotypes from EHR data automatically, can accelerate the adoption and utilization of phenotype-driven efforts to advance scientific discovery and improve healthcare delivery. A list of computational phenotyping algorithms has been published but data fragmentation, i.e., incomplete data within one single data source, has been raised as an inherent limitation of computational phenotyping. In this study, we investigated the impact of diverse data sources on two published computational phenotyping algorithms, rheumatoid arthritis (RA) and type 2 diabetes mellitus (T2DM), using Mayo EHRs and Rochester Epidemiology Project (REP) which links medical records from multiple health care systems. Results showed that both RA (less prevalent) and T2DM (more prevalent) case selections were markedly impacted by data fragmentation, with positive predictive value (PPV) of 91.4 and 92.4%, false-negative rate (FNR) of 26.6 and 14% in Mayo data, respectively, PPV of 97.2 and 98.3%, FNR of 5.2 and 3.3% in REP. T2DM controls also contain biases, with PPV of 91.2% and FNR of 1.2% for Mayo. We further elaborated underlying reasons impacting the performance.

## Introduction

The increased availability of EHRs fostered by the HITECH Act has a great potential to serve as a rich, integrated source of phenotype information (Denny et al., 2011; Crawford et al., 2014). Critical to this effort is computational phenotyping, which identifies patients with certain conditions of interest from EHR data (Gunasekar et al., 2016). A list of computational phenotyping algorithms covering over fifty diseases (PheKB, 2019) including RA (Liao et al., 2010; Partners Phenotyping Group, 2016) and T2DM are available at the PheKB, primarily developed through the eMERGE Network. The majority of the eMERGE phenotyping algorithms were developed to identify cases and controls of specific medical conditions for use in genome and phenome-wide association studies (Ritchie et al., 2010). However, developing research quality EHR-based computational phenotyping algorithms that categorize disease or traits in complete populations is not an easy task, as the primary purpose of EHR data is for healthcare delivery and reimbursement practices (Wei and Denny, 2015). Case/control EHR algorithms are powerful tools in research, however, the ability to characterize real-world clinical patient populations that are comprised of a mix of primary care patients (i.e., medical home), transient patients, and referral patients resulting in varying patterns of depth and detail in EMR data is more challenging. Thus, data fragmentation, or incomplete data due to patient movement across healthcare institutions, is an inherent limitation of using EHR data for research (Robinson et al., 2018), and creates challenges in validating EHR-based phenotypes in populations (Newton et al., 2013). While extensive investigations on computational phenotyping have been performed to improve algorithm performance and portability (Kullo et al., 2010; Kurreeman et al., 2011; Newton et al., 2013; Wei and Denny, 2015; Teixeira et al., 2016), the impact of data fragmentation on computational phenotyping is under investigated. We identified only one study which evaluated the impact of data fragmentation on algorithm performance. Using data from two healthcare institutions, the study demonstrated that running a T2DM phenotyping algorithm, developed by researchers from Northwestern University, on data from a single institution missed almost one third of the T2DM cases (Wei et al., 2012).

Using the Mayo Biobank cohort, we assessed the impact of data fragmentation on two popular eMERGE phenotyping algorithms, RA and T2DM, as these two diseases differ greatly in prevalence. Specifically, the overall prevalence of RA is estimated at 0.5%(Hunter et al., 2017) while T2DM is a common disease affecting approximately 8.6% adults in the United States (Bullard et al., 2018). Additionally, the RA algorithm is regression-based while the T2DM algorithm is rule-based.

## Materials and Methods

### Data Sources

In this study, we used the Mayo Clinic Biobank cohort (Olson et al., 2013) with the following self-reported data collected at the time of consent into the Biobank: general health, self and first-degree relative family disease history, and demographic characteristics. The clinical data for the cohort can be retrieved from two sources: Mayo Clinic EHRs and the REP (Rocca et al., 2018). The REP is a record linkage system which links and archives medical records from multiple healthcare providers in Minnesota and Wisconsin including Mayo Clinic since 2010 (Rocca et al., 2018). All analyses were based on a subset of the Biobank cohort consisting of 45,183 patients who have at least one diagnosis code in Mayo Clinic EHRs and at least one diagnosis code in the REP during 2010 and 2017. We used diabetes family history from the Biobank self-reported data to run the T2DM phenotyping algorithm. All patients in the chosen Biobank cohort consented to have their EHR data used for research. This study was approved by the Institutional Review Board of Mayo Clinic.

### Phenotyping Algorithms

**FIGURE 1:**
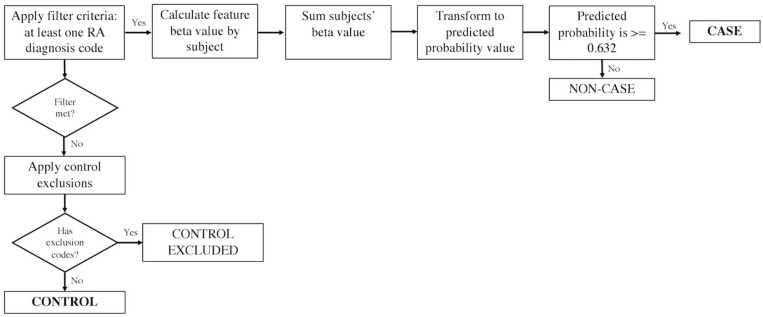
The eMERGE algorithm for identifying RA cases and controls. Adapted from Partners Phenotyping Group (2016).

The eMERGE RA algorithm was created using a machine-learning penalized logistic regression model trained on a screen-positive data set with at least one RA diagnosis code (inclusion cohort) (Figure 1). Among the training set, the gold standard for model development was built up based on the 2010 American College of Rheumatology criteria for classification of RA (Aletaha et al., 2010). In the model, relative weights for features significantly associated with RA, including diagnosis of RA, SLE, PA, lab tests for rheumatoid factor, and total number of encounters (visits) per subject were assigned. Once the model was created, a threshold value based on a specificity of 97% was selected to identify cases. We used an updated version of the algorithm (the Harvard eMERGE RA Algorithm Document downloaded from https://phekb.org/phenotype/rheumatoid-arthritis-ra) which incorporates ICD 9 and 10 codes. Comparing to the previous version using only ICD 9 (Carroll et al., 2012), the updated version achieved sensitivity, PPV, and overall area under the curve of 87, 95, and 95%, respectively, compared to 65, 90, and 95% previously. Controls were selected from persons without any RA diagnosis codes and without any exclusion codes.

**FIGURE 2:**
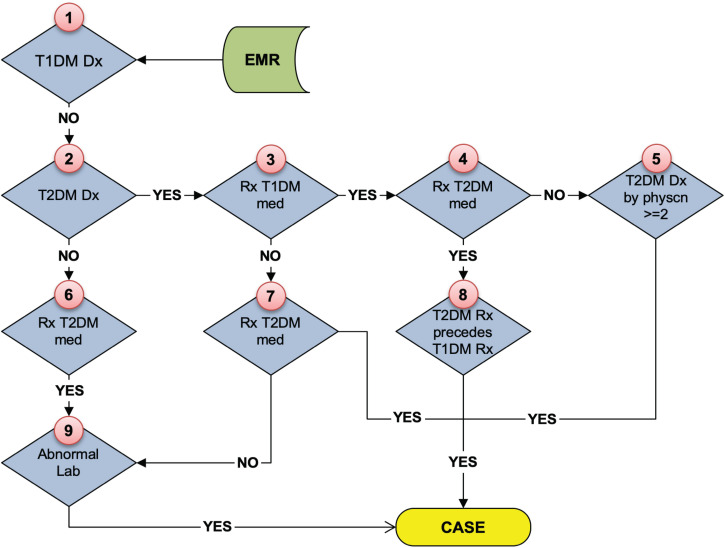
The eMERGE algorithm for identifying T2DM cases. Adapted from Pacheco and Thompson (2012).

**FIGURE 3:**
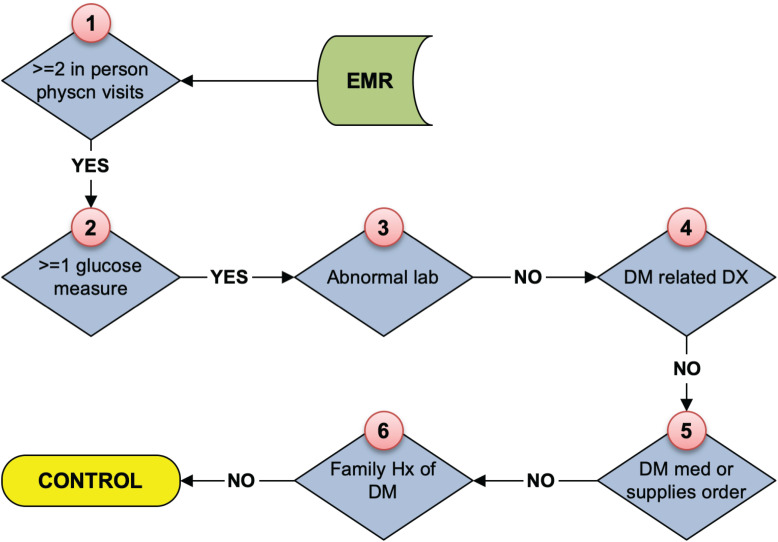
The eMERGE algorithm for identifying T2DM controls. Adapted from Pacheco and Thompson (2012).

As stated above, the eMERGE T2DM algorithm^1^ was developed by researchers from Northwestern University in 2012 (Kho et al., 2011). It is a rule-based algorithm based on diabetes related diagnosis, lab, and medication information, achieving 98 and 100% PPV for T2DM case and control identification, respectively. In addition to structured EHR data, in this study, we also extracted physician entered diagnoses from clinical notes of both Mayo and REP sources leveraging natural language processing (NLP) techniques. We updated the algorithm to incorporate ICD 10 codes which have been adopted in late 2015 in the United States (Hirsch et al., 2016). To collect more complete data, we also added medication extracted by NLP from clinical notes in both Mayo and REP sources. Figure 2 shows the flowchart of T2DM case phenotyping algorithm and Figure 3 shows the flowchart of T2DM control phenotyping algorithm.

### Analysis

We assessed data fragmentation by evaluating the performance of the phenotyping algorithms in the cohort. To evaluate phenotyping errors caused by data fragmentation across health institutions, we used subjects identified from the combination of Mayo and REP data as the benchmark. To evaluate the performance of the phenotyping algorithms, we randomly selected 50 cases and 50 controls for each algorithm from the benchmark to perform manual review. We calculated sensitivity, specificity, PPV and FNR based on the number of TP, FP, TN, and FN against the benchmark (Wei et al., 2012). The impact of various data sources on quantitative change of features contributing to phenotyping was analyzed.

## Results

Table 1 shows the number of cases and controls identified by each data source as well as the combination of sources. Using both Mayo Clinic EHRs and REP data, we identified 620 RA cases (42,319 controls) and 5,215 T2DM cases (6,293 controls) to serve as our benchmark for the analyses. Table 2 shows performance of RA and T2DM phenotyping algorithms in benchmark against chart reviewed gold standards. PPV of RA case was 90% compared to 95% in the updated version of the algorithm. PPVs of T2DM case and control were 82 and 100% compared to 98 and 100% in the publication (Kho et al., 2011).

**TABLE 1:** Phenotyping results using various data sources.

**Phenotyping algorithm**	**Case**			**Control**		
	**Mayo+REP**	**REP**	**Mayo**	**Mayo+REP**	**REP**	**Mayo**
Rheumatoid Arthritis	620	605	498	4,2319	42,398	43,070
Type 2 diabetes mellitus	5,215	5,124	4,850	6,293	6,482	6,815

**TABLE 2:** Performances of RA and T2DM phenotyping algorithms in benchmark (against chart reviewed gold standard).

**Phenotyping algorithms**	**TPs**	**FPs**	**TNs**	**FNs**	**Sensitivity (TP/TP+FN), %**	**Specificity [TN/(FP+TN)], %**	**PPV [TP/(TP+FP)], %**	**FNR [FN/(TP+FN)], %**
RA case	45	5	49	1	97.8	90.7	90.0	2.2
RA control	49	1	45	5	90.7	97.8	98.0	9.3
T2DM case	41	9	50	0	1	84.7	82.0	0
T2DM control	50	0	41	9	84.7	1	1	15.3

Table 3 shows benchmark performance of RA and T2DM cases and controls using various data sources. Using Mayo only data, the RA phenotyping algorithm identified 43 FP RA case subjects, missed 165 FN RA case subjects (FNR, 26.6%), and identified 751 FP RA control subjects because of data fragmentation across healthcare institutions. The T2DM phenotyping algorithm identified 368 FP T2DM case subjects, missed 733 FN T2DM case subjects (FNR, 14%), identified 597 FP T2DM control subjects and missed 75 FN T2DM control subjects (FNR, 1.2%) because of data fragmentation across healthcare institutions. Fewer errors were resulted using REP only data, the RA phenotyping algorithm identified 17 FP RA case subjects, missed 32 FN RA case subjects (FNR, 5.2%), and identified 79 FP RA control subjects because of data fragmentation across healthcare institutions. The T2DM phenotyping algorithm identified 91 FP T2DM case subjects, missed 173 FN T2DM case subjects (FNR, 3.3%), identified 245 FP T2DM control subjects and missed 56 FN RA control subjects (FNR, 0.9%) because of data fragmentation across healthcare institutions.

**TABLE 3:** Benchmark performance of RA and T2DM cases and controls using various data sources.

**Phenotyping algorithm**		**Data source**	**TPs**	**FPs**	**TNs**	**FNs**	**Sensitivity (TP/TP+FN), %**	**Specificity [TN/(FP+TN)], %**	**PPV [TP/(TP+FP)], %**	**FNR [FN/(TP+FN)], %**
RA	Case	Mayo+REP	620	0	44,563	0	100	100	100	0
		Mayo	455	43	44,520	165	73.4	99.9	91.4	26.6
		REP	588	17	44,546	32	94.8	99.9	97.2	5.2
	Control	Mayo+REP	42,319	0	2,864	0	100	100	100	0
		Mayo	42,319	751	2,113	0	100	73.8	98.3	0
		REP	42,319	79	2,785	0	100	100	99.8	0
T2DM	Case	Mayo+REP	5,215	0	39,968	0	100	100	100	0
		Mayo	4,482	368	39,600	733	86.0	99.1	92.4	14.0
		REP	5,124	91	39,795	173	96.7	99.8	98.3	3.3
	Control	Mayo+REP	6,293	0	38,890	0	100	100	100	0
		Mayo	6,218	597	38,293	75	98.8	98.5	91.2	1.2
		REP	6,237	245	38,645	56	99.1	99.4	96.2	0.9

**FIGURE 4:**
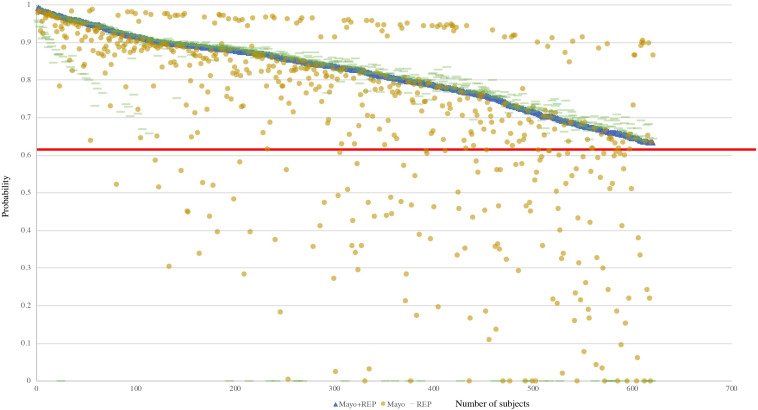
Probability of all benchmark cases based on various data sources. The red line intercepts the cutoff of 0.632, probabilities above the red line are classified as RA cases.

Further analysis showed that the 150 out of 165 RA false negative cases obtained from the Mayo only data source were in the Mayo Clinic inclusion cohort (positive-screening set; Table 3). Among the 32 RA false negative cases in REP data source, all were in the REP inclusion cohort. Figure 4 presents the RA probabilities of all benchmark cases (620) derived from Mayo Clinic, REP, and the combination of Mayo and REP data. Probability estimated from Mayo and REP data show multiple regression lines, reflecting heterogenicity among healthcare providers. The red line intercepts the cutoff of 0.632; probabilities above the red line indicate RA cases. Figure 4 shows that probabilities of the FN in Mayo or REP sources improved after combining Mayo data with REP data.

Table 4 shows statistics of features associated with RA phenotyping in various data sources between 2010 and 2017, specifically the number of patients with RA, PA, and SLE diagnosis codes, laboratory tests for rheumatoid factor as well as total number of encounters for the Mayo Biobank cohort. The inclusion cohort increased to 2,127 using both Mayo and REP data, with an increase of 524 compared to Mayo and 69 compared to REP. 15 of the 524 was identified as an RA case in the combined Mayo and REP data. Supplementary Figure S1 shows RA case probabilities of the false negative Mayo inclusion cohort (524 subjects) in the combination of Mayo and REP data, only several subjects had values close to the cutoff (0.632). Supplementary Figure S2 shows RA case probabilities of the false negative REP inclusion cohort (69 subjects) in the combination of Mayo and REP data, no subject had value above the cutoff.

**TABLE 4:** Statistics of features associated with RA case phenotyping in various data sources.

**Data sources**	**# of Cases meeting algorithm criteria**	**# of patients with 1+ RA diagnosis codes (inclusion cohort)**	**# of Patients with 1+ PA diagnosis codes**	**# of patients with 1+ SLE diagnosis codes**	**# of patients with 1+ Lab test for rheumatoid factor**	**Total encounter No. (for the Mayo Clinic Biobank cohort)**
Mayo+REP	620	2,127	83	68	1,293	3,039,483
Mayo	498	1,603	52	53	907	2,850,109
REP	605	2,058	79	64	1,254	2,776,569

*RA, rheumatoid arthritis; PA, psoriatic arthritis; SLE, systemic lupus erythematosus.*

**FIGURE 5:**
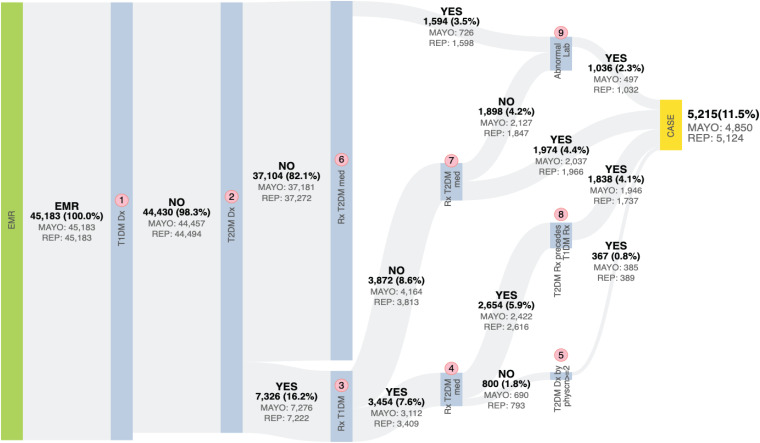
Quantitative comparison of each step in T2DM case phenotyping among various data sources. The number of each step corresponds to Figure 2, bold numbers are derived from the combination of Mayo of REP data.

**FIGURE 6:**
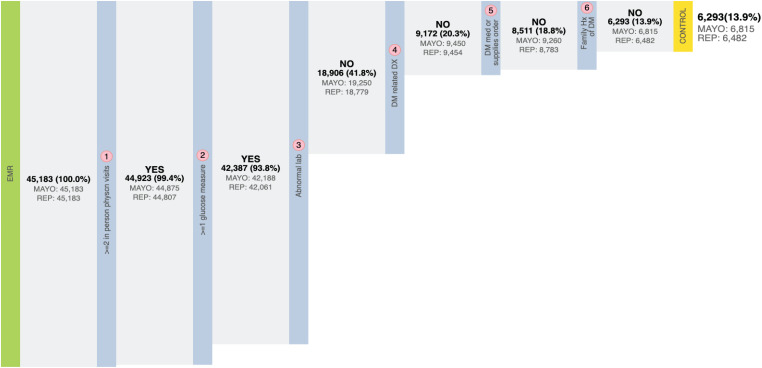
Quantitative comparison of each step in T2DM control phenotyping among various data sources. The number of each step corresponds to Figure 3, bold numbers are derived from the combination of Mayo of REP data.

The flowcharts (Figures 5, 6) show the comparison of each step of T2DM case and control phenotyping algorithms among various data sources, where the number denoting each step is from Figures 2, 3, respectively. The detailed statistics are provided in Supplementary Tables S1, S2. Using only Mayo data, 1 of the 733 FN T2DM cases have been falsely identified as a control, and 1 benchmark case was falsely identified as a control among the 597 FP T2DM controls. Table 5 shows factors that contribute to FP and FN RA case subjects. There are 751 FP controls from Mayo and 79 FP controls from REP due to incomplete diagnosis codes in each institution. Table 6 shows factors that contribute to FP and FN T2DM case subjects. Table 7 shows factors that contribute to FP and FN T2DM control subjects.

**TABLE 5:** Missing information for FN and FP RA case subjects.

**Error type**	**Data sources**	**Total No. of subjects**	**No. of subjects with missing information**				
			**# of patients with 1+ RA diagnosis codes (inclusion cohort)**	**# of Patients with 1+ PA diagnosis codes**	**# of patients with 1+ SLE diagnosis codes**	**# of patients with 1+ Lab test for rheumatoid factor**	**Total encounter No.**
FN	Mayo	165	15	1	0	14	17,674
	REP	32	0	0	0	0	2,654
FP	Mayo	43	0	0	0	0	2,112
	REP	17	0	0	0	0	1,083

**TABLE 6:** Missing information for FN and FP T2DM case subjects.

**Error type**	**Data source**	**Total No. of subjects**	**No. of subjects with missing information**					
			**T1DM Dx**	**T2DM Dx**	**T1DM drug**	**T2DM drug**	**Abnormal lab**	**T2DM Dx by physician**
FN	Mayo	733	0	22	36	248	532	0
	REP	173	0	46	3	25	8	0
FP	Mayo	368	246	17	90	39	25	0
	REP	82	0	12	13	7	0	0

**TABLE 7:** Missing information for FN and FP T2DM control subjects.

**Error type**	**Data source**	**Total No. of subjects**	**No. of subjects with missing information**					
			**≥2 in person physician visits**	**≥1 glucose measure**	**Abnormal lab**	**DM related Dx**	**DM med or supplies order**	**Family Hx of DM**
FN	Mayo	75	10	65	0	0	0	0
	REP	56	11	45	0	0	0	0
FP	Mayo	597	0	0	122	166	362	0
	REP	245	0	0	4	236	5	0

## Discussion

Electronic health records have been an asset for studies whose goal is to define cases and controls with high accuracy. However, leveraging EHRs for population studies is more challenging. Inherent data issues and bias (e.g., data fragmentation and referral bias) of EHRs may result in inaccurate and biased phenotyping results. RA and T2DM phenotyping algorithms to define cases and controls have been well studied by researchers with high performances within separate institutions (Carroll et al., 2012; Wei et al., 2012). These two diseases have also been fertile ground for secondary use of EHRs in combination with DNA samples (Wood et al., 2008; Kurreeman et al., 2011; Carroll et al., 2015). Therefore, it is worthwhile to investigate and evaluate the impact of data fragmentation on completeness and validity of algorithm results. To the best of our knowledge, this study is the first to evaluate the impact of data fragmentation on RA phenotyping algorithm. When assessing the impact of data fragmentation on T2DM, the FNR is 14% in our current study in comparison to 33% reported previously (Wei et al., 2012) as we leveraged NLP to extract more complete diagnosis and medication information from clinical notes. Our performance metrics of the benchmark against chart reviewed gold standard are slightly inferior to those metrics published previously, which is consistent with other studies, primarily due to the heterogenicity of healthcare systems impacting algorithm portability (Ostropolets et al., 2020).

As a regression-based algorithm, the sensitivity of the RA phenotyping depends on the comprehensiveness of the data, partially due to the complex relationships between autoimmune disorders. Systemic lupus erythematosus and PA both interfere with RA diagnosis. Total encounter numbers also exert adverse effects on RA case identification. Though RA diagnosis and positive RA lab results were assigned with relatively high positive weights, SLE, PA, and all encounter numbers were assigned with negative weights in the algorithm. In our study, 150 out of 165 FN cases in Mayo were already in the Mayo inclusion cohort and all FN cases in REP were in the REP inclusion cohort.

As a medical record-linkage system, REP captures more comprehensive diagnosis codes than Mayo EHRs resulting in fewer benchmark phenotyping errors. Meanwhile, not all information in Mayo EHRs is included in REP. For example, REP misses the diagnosis information coming from the Problem List in Mayo EHRs. Using Mayo only data, the RA phenotyping algorithm missed 165 FN RA case subjects (FNR = 26.6%) and the T2DM phenotyping algorithm missed 733 FN T2DM case subjects (FNR = 14%). All FN and FP cases, FN and FP controls defined using only Mayo data or only REP data were masked or falsely identified due to missing or insufficient information.

Although we investigated only two phenotyping algorithms in this study, the findings would also have implications for other research that relies on case and control identification using EMERGE algorithms. Considering the potential impact of data fragmentation on data quality for genomic research, clinical researchers should always keep this caveat in mind when employing a phenotyping algorithm for such studies. Ideally, adding data from a medical record linkage system or a comprehensive claims data source (such as the Centers for Medicare and Medicaid Services) can capture and reuse clinical information more efficiently. In addition, unstructured data extracted using NLP techniques could help to decrease data fragmentation because unstructured clinical notes in EHRs often record patients’ history of past diseases which may have been originally diagnosed and treated in other health institutions. Finally, besides the data fragmentation issue, temporal issues could also result in incomplete phenotyping results. Researchers have conducted a related study on T2DM (Wei et al., 2013), and we also investigated the dependence of the eMERGE RA algorithm on both RA and electronic health record (EHR) duration in another manuscript (Journal of the American Medical Informatics Association, in press).

The limitation of the study is that we didn’t have fully-annotated gold standards. Because of the high PPV seen in previous studies (Kho et al., 2011; Carroll et al., 2012), (95% for RA case, 98% and 100% for T2DM case and control), we set the benchmark for evaluation to be the phenotyping results based on the combination of data from both Mayo and REP, and only manually reviewed 50 cases and 50 controls for each algorithm to validate. In addition, the study would have been more generalizable if data from multiple sites had been independently collected.

## Conclusion

In this study, we demonstrated that various data sources may result in different phenotyping results through two case studies. We also identified underlying reasons for variation in the algorithm performance. The completeness and validity of phenotypic and exposure information derived from EHRs are the foundations for precision medicine and patient care. As more genomic research makes use of EHR-derived phenotypes, it is important to understand that data fragmentation may significantly affect algorithm performance.

## Data Availability Statement

The datasets for this article are not publicly available because: The Ethical approval is contingent on the data remaining private. Requests to access the datasets should be directed to HL, liu.hongfang@mayo.edu.

## Ethics Statement

The studies involving human participants were reviewed and approved by the Institutional Review Board (IRB) of Mayo Clinic. The Ethics Committee waived the requirement of written informed consent for participation.

## Author Contributions

All co-authors are justifiably credited with authorship, according to the authorship criteria. All authors read and approved the final manuscript. LW: design, development, data collection, analysis of data, interpretation of results, drafting and revision of the manuscript. MH, SF, and HH: data collection. JO, SB, JS, MC, and JC: critical revision of the manuscript. HL: conception, design, analysis of data, interpretation of results, critical revision of the manuscript.

## Conflict of Interest

The authors declare that the research was conducted in the absence of any commercial or financial relationships that could be construed as a potential conflict of interest.

## Footnotes

1 https://phekb.org/phenotype/type-2-diabetes-mellitus

## Abbreviations

EHRs: electronic health records
eMERGE: electronic medical records and genomics
FN: false negatives
FNR: false-negative rate
FP: false positives
HITECH: health information technology for economic and clinical health
PA: psoriatic arthritis
PheKB: phenotype knowledgebase
PPV: positive predictive value
RA: rheumatoid arthritis
REP: rochester epidemiology project
SLE: systemic lupus erythematosus
T2DM: type 2 diabetes mellitus
TN: true negatives
TP: true positives
