# Supplementary figures and images for: Impact of Diverse Data Sources on Computational Phenotyping
Source: Front Genet. 2020 Jun 3;11:556. doi: 10.3389/fgene.2020.00556 (PMC7283539; doi:10.3389/fgene.2020.00556)

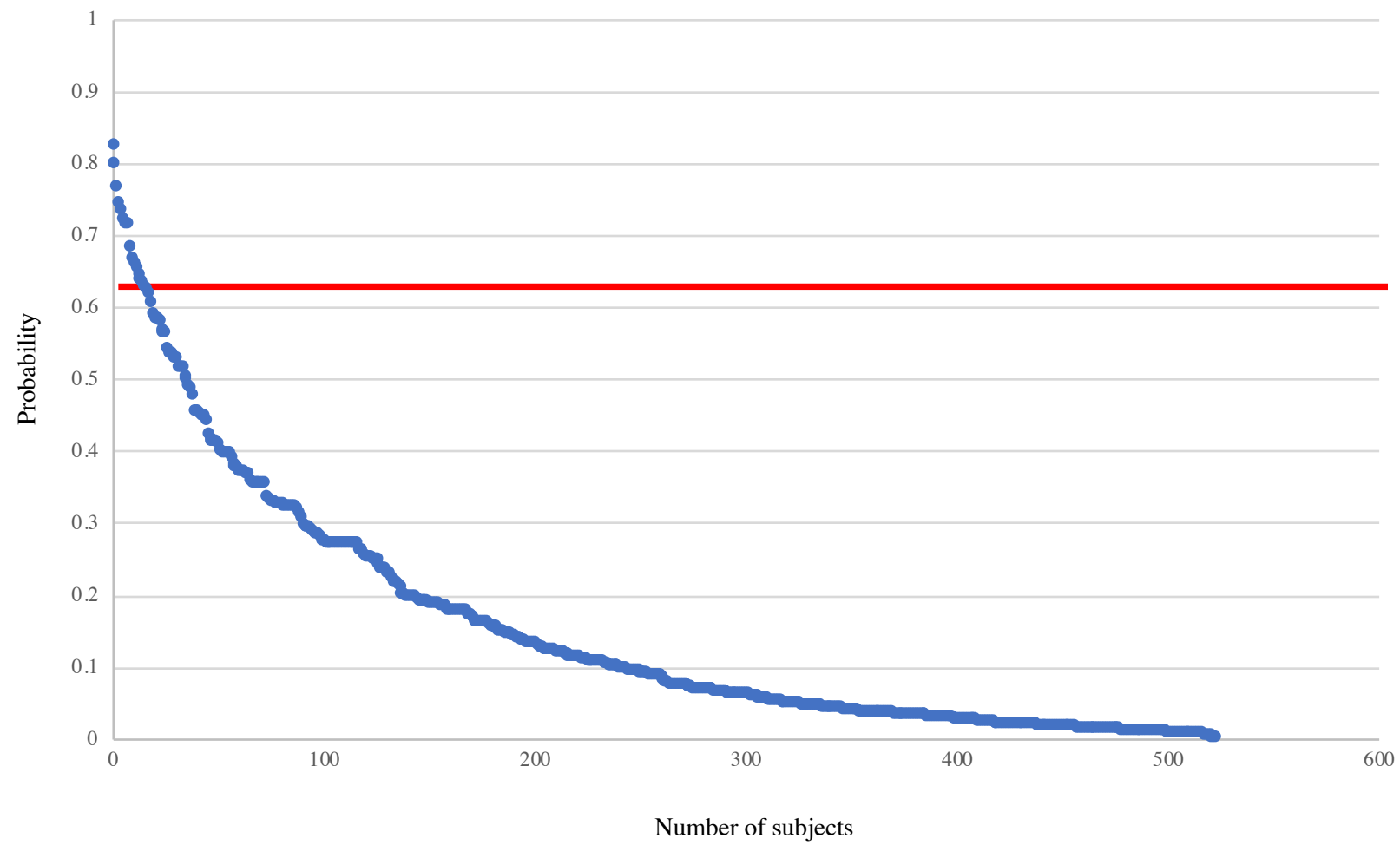

Supplement: Supplementary file 1 [file Image_1.pdf]

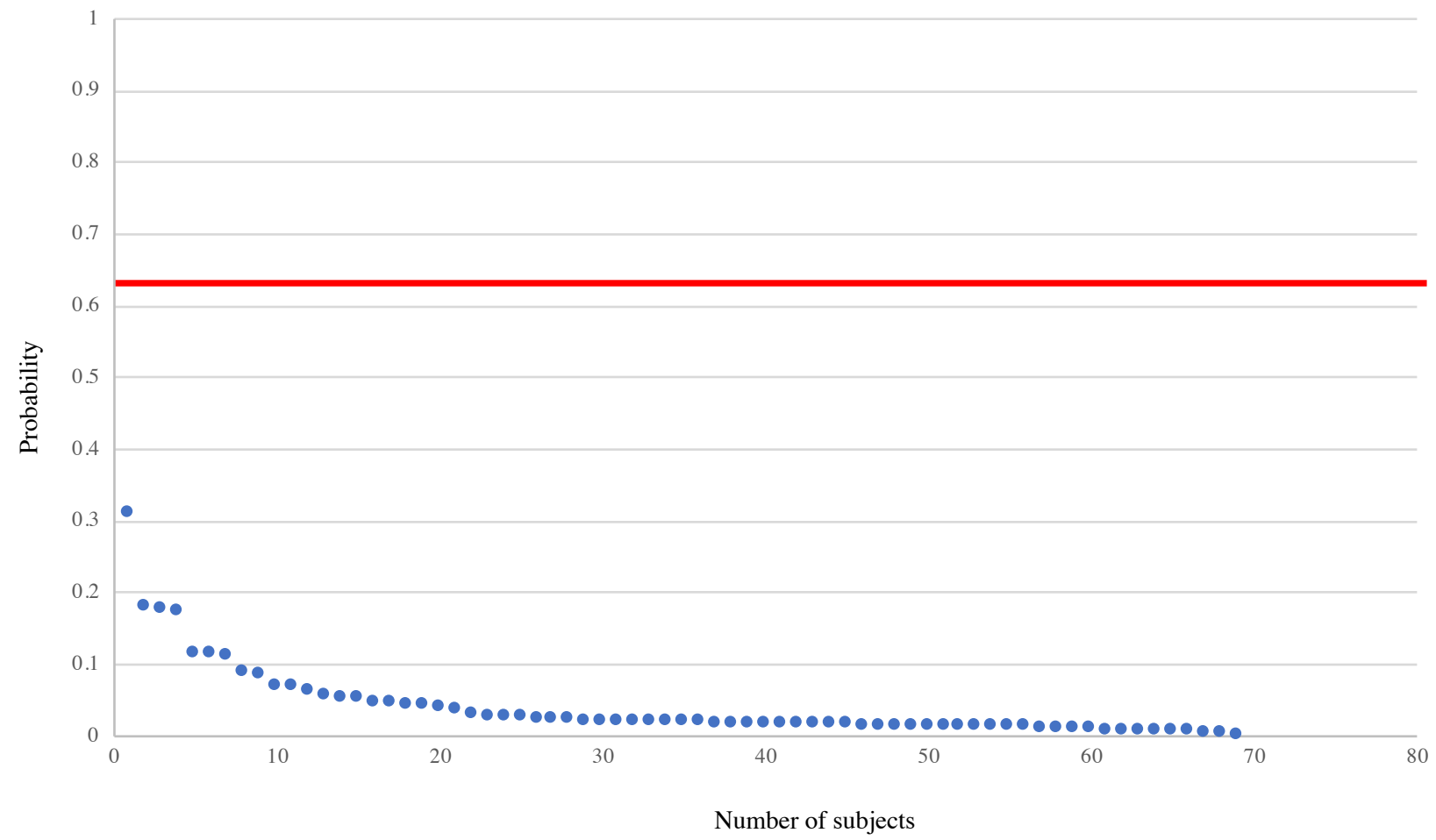

Supplement: Supplementary file 2 [file Image_2.pdf]
