# Supplementary material for: Impact of Diverse Data Sources on Computational Phenotyping
Source: Front Genet. 2020 Jun 3;11:556. doi: 10.3389/fgene.2020.00556 (PMC7283539; doi:10.3389/fgene.2020.00556)
Supplement: Supplementary file 3 [file Table_1.docx]

**Supplemental Table 1. Statistics of features associated with T2DM case phenotyping**

|  | No. of cohort with EHR | T1DM Dx | T2DM Dx | T1DM drug | T2DM drug | Abnormal lab | T2DM Dx by physician |
| --- | --- | --- | --- | --- | --- | --- | --- |
| Mayo | 45,183 | 726 | 7,276 | 5,041 | 5,485 | 6,406 | 4,417 |
| REP | 45,183 | 689 | 7,222 | 7,079 | 6,497 | 8,782 | 4,420 |
| Mayo+REP | 45,183 | 753 | 7,402 | 7,171 | 6,578 | 8,822 | 4,420 |
