# Supplementary material for: Impact of Diverse Data Sources on Computational Phenotyping
Source: Front Genet. 2020 Jun 3;11:556. doi: 10.3389/fgene.2020.00556 (PMC7283539; doi:10.3389/fgene.2020.00556)
Supplement: Supplementary file 4 [file Table_2.docx]

**Supplemental Table 2. Statistics of features associated with T2DM control phenotyping**

|  | No. of cohort with EHR | >=2 in person physcn visits | >=1 glucose measure | Abnormal lab | DM related Dx | DM med or supplies order | Family Hx of DM | Control |
| --- | --- | --- | --- | --- | --- | --- | --- | --- |
| Mayo | 45,183 | 44,875 | 42,285 | 22,950 | 25,453 | 7,776 | 18,572 | 6,815 |
| REP | 45,183 | 44,807 | 42,166 | 23,304 | 26,756 | 10,379 | 18,572 | 6,482 |
| Mayo+REP | 45,183 | 44,923 | 42,461 | 23,491 | 28,051 | 10,472 | 18,572 | 6,293 |
